# Efficient energy and completion time for dependent task computation offloading algorithm in industry 4.0

**DOI:** 10.1371/journal.pone.0252756

**Published:** 2021-06-08

**Authors:** Rabab Farouk Abdel-Kader, Noha Emad El-Sayad, Rawya Yehia Rizk

**Affiliations:** Electrical Engineering Dept., Port Said University, Port Said, Egypt; Vellore Institute of Technology: VIT University, INDIA

## Abstract

Rapid technological development has revolutionized the industrial sector. Internet of Things (IoT) started to appear in many fields, such as health care and smart cities. A few years later, IoT was supported by industry, leading to what is called Industry 4.0. In this paper, a cloud-assisted fog-networking architecture is implemented in an IoT environment with a three-layer network. An efficient energy and completion time for dependent task computation offloading (ET-DTCO) algorithm is proposed, and it considers two quality-of-service (QoS) parameters: efficient energy and completion time offloading for dependent tasks in Industry 4.0. The proposed solution employs the Firefly algorithm to optimize the process of the selection-offloading computing mode and determine the optimal solution for performing tasks locally or offloaded to a fog or cloud considering the task dependency. Moreover, the proposed algorithm is compared with existing techniques. Simulation results proved that the proposed ET-DTCO algorithm outperforms other offloading algorithms in minimizing energy consumption and completion time while enhancing the overall efficiency of the system.

## I. Introduction

The tremendous evolution of the Internet of Things (IoT) arises from the fact that massive sensors are interconnected to IoT technologies where these sensors produce enormous amounts of data and requests.

In a smart industry, devices are interconnected with data sensing and computing capabilities, thereby shaping Industry 4.0, which refers to the fourth industrial revolution. IoT, fog computing, cloud computing [1], and other advanced technologies can be used to provide Industry 4.0 with a fully connected smart network to reduce energy consumption and task computation time while enhancing production. In a smart factory, IoT connects machines, humans, and things [2]. Environmental, equipment, and personal information are gathered from intelligent terminal devices, such as sensors, mobile, and smart devices. Cloud provides data processing and analysis services, and cloud computing provides a stable base for intelligent manufacturing implementation [3]. Because of the enormous development of IoT and Industry 4.0, applications for terminal devices send large amounts of data to a cloud; thus, congestion and bottleneck problems occur, which prevent the cloud to meet the requirements of quality-of-service (QoS) [4].

In a smart factory, cloud computing has problems and challenges due to intelligent manufacturing processes that increase the number of computation-intensive and delay-sensitive tasks to satisfy the small-batch production of individualized goods [5]. For cloud computing, providing real-time knowledge of the state of working equipment is not easy. Production scheduling in a smart factory is a multi-object and multitask application in real-time. A cloud has problems in ensuring QoS, such as real-time efficiency and reliability. Thus, fog computing is implemented in a smart factory at the network’s fog layer, and because it is closest to the terminal sensors, it enables the user to offload data to fog or cloud servers with low latency, low energy consumption, and high reliability [6–9].

Three computing modes exist for each terminal sensor: local, fog, and cloud computing. In the IoT scheme, tasks can be categorized into local and offloaded. The local tasks are computed locally via terminal devices because they are difficult real-time tasks, such as fire alarms or an automatic shut off the boiler valve. If these tasks are delayed, tragic failure or a loss of life may occur. The offloaded tasks are the tasks that fog, or cloud servers can handle. In this paper, a mixed computing model is investigated.

**Fig 1:**
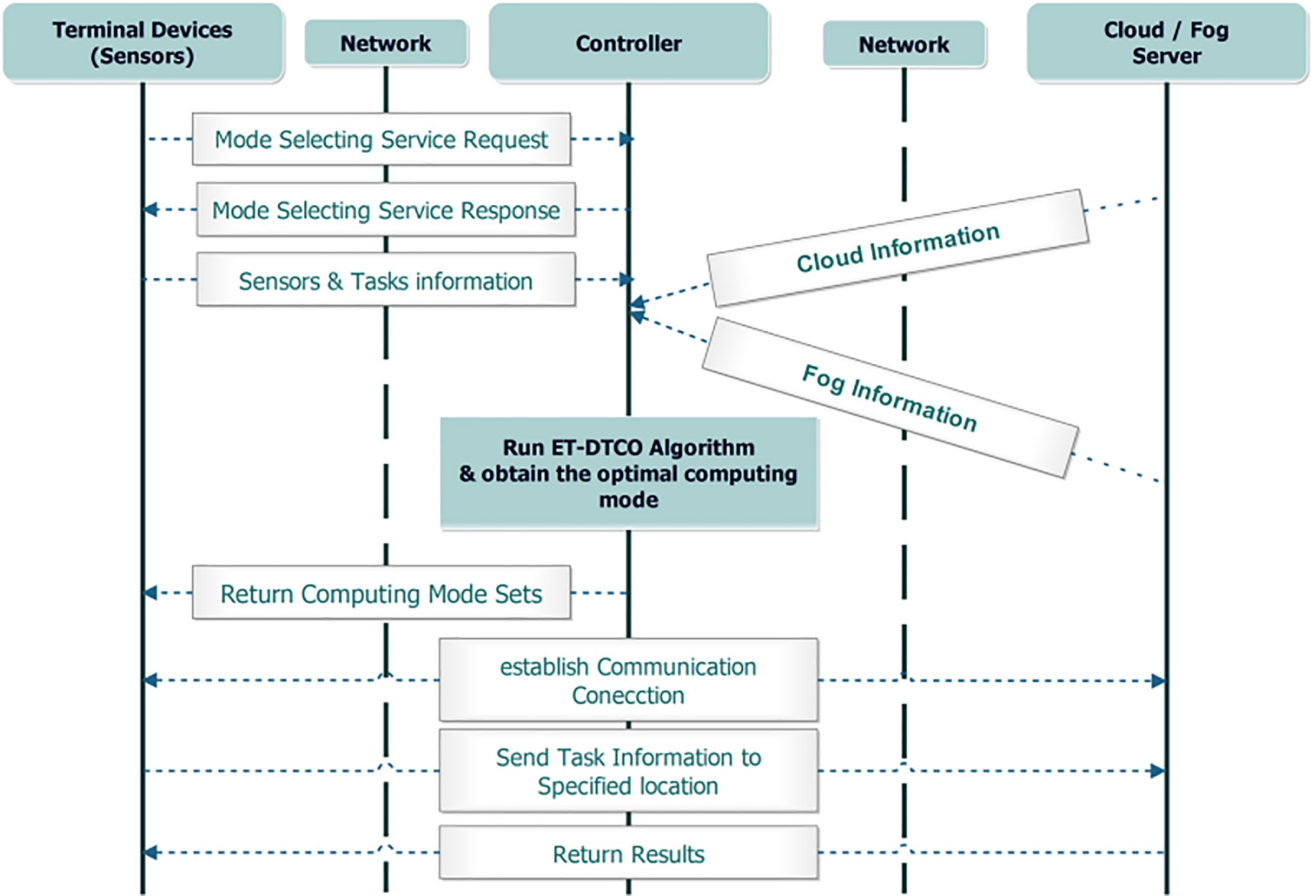
Workflow diagram.

The proposed algorithm determines the optimal computing model for dependent tasks to satisfy the minimum energy consumption and low completion time using Firefly (FF) algorithm [10,11], which is a meta-heuristic optimization algorithm [12] with many rules to solve single or multi-target problems based on swarm intelligence. The algorithm has a global search capability with Levy flight’s process which eliminates the randomness of iteration for finding the best solution to a given problem [13]. The workflow diagram is shown as Fig 1.

The proposed offloading algorithm selects among servers similar to the FF algorithm, with a modification to the attractive function to calculate the attractiveness of FF (computational server) instead of distance, and deciding automatically the optimal offloading model using the updated objective function and brighter fireflies (optimal computational server as a type of fog or cloud servers). This resulting in an efficient energy and completion time computation offloading (ET-DTCO) algorithm for dependent task.

The main contribution of the proposed algorithm can be summarized as proposing an offloading strategy for dependent tasks in Industry 4.0 taking into consideration two QoS parameters, namely, energy consumption and completion time. To evaluate the quality of the solution, a weighted sum approach-based fitness function is used to deal with the objective aspects. The paper implements a modified FF algorithm to address the optimal three—layer offload strategy for each task, then selects the optimal computational mode for each task. The paper presents an analytical framework to perform a comprehensive analysis of ET-DTCO algorithm. It compares the proposed algorithm with state-of-the-art competing techniques.

The remainder of this paper is organized as follows. Section II introduces research works on related issues. The system model and technical background regarding communication, computation, and task dependence models are presented in Section III. In Section IV, we discuss the expressed offloading system and FF optimization algorithm. Numerical results are evaluated in Section V. Finally, Section VI concludes the study.

## II. Related work

With the exponential development of data size, the computational offloading process in fog or cloud computing is an essential issue for further improving service quality and energy consumption.

Computational offloading algorithms are classified into three categories. The first category of algorithms [14–16] examined computational offloading optimization based on one performance indicator, such as energy consumption or latency time. Le et al. [14] proposed an efficient resource allocation in the computation offloading of the mobile-edge algorithm by jointly allocating radio and computing resources to achieve the optimal offloading scheme of the task in minimum completion time. Wang et al. [15] used the software-defined network-based industrial IoT with fog computing based on computing mode selection and completing sequences that can be determined by task priority. This algorithm satisfies real-time performance in offloading computing optimization. From the energy consumption perspective, Zhao et al. [16] developed an energy-efficient computing-offloading system in the fog that allows a device-to-device (D2D) network by expressing the problem of energy consumption reduction. Although these algorithms achieved performance gains at the energy consumption or latency time, they cannot satisfy the expected energy consumption with the least or expected completion time with the minimum energy consumption.

The key concept of the second category of algorithms [17–19] is to reduce energy consumption with a defined latency constraint or combined latency and energy constraints. Chen et al. [17] proposed an accelerated gradient algorithm to achieve the minimum energy consumption of computation tasks within the desired energy overhead and delay at the fog server. These approaches required implementing the green framework [20] and deep learning [21] algorithms to achieve a smart and green offloading decision. However, Meng et al. [18] suggested a system to reduce the total energy consumption with a constraint on task offloading delay. This method applied constraint in a hybrid fog and cloud framework. Wang et al. [19] improved the latency and energy consumption by employing task offloading and computing in wireless powered by mobile-edge computing systems. This category enhanced the performance of the first category; however, it did not address the problem of the mutual optimization of energy consumption and completion time, which could significantly improve the effectiveness of the entire system.

To improve the performance of computing systems, the third category of algorithms [22–25] achieved the optimization of two or more performance indicators simultaneously. Du et al. [22] optimized the problem of minimizing energy consumption and latency in a mixed system of fog and cloud. Dinh et al. [23] achieved the joint optimization problem of the computational delay of tasks and energy usage of mobile devices in mobile-edge computing. From the reliability and latency viewpoint, J. Liu et al. [24] developed a joint optimization problem to simultaneously decrease the amount of offloading letdown and completion time of tasks. These algorithms have developed a multi-objective computational latency, energy consumption, and overhead optimization system for fog-computing offloading. L. Liu et al. [25] solved this multi-objective minimization problem by discovering the optimum transmission power and possibility of offloading. Chang et al. [26] suggested an alternating direction method of multipliers based distributed (ADMMD) algorithm to solve the problem of minimizing energy consumption by meeting the delay constraint. However, they did not consider the dependencies among tasks in the IoT sensor nor the coordination between fog and cloud to minimize energy consumption and task completion time.

Sun et al. [27] and F. Liu et al. [28] suggested energy and time-efficient computation offloading and resource allocation (ETCORA) and energy-efficient collaborative task computation offloading (ECTCO) algorithms, respectively, to solve the problem of minimizing the energy consumption and completion time of requests by cooperation between the fog and cloud wherein the fog did not replace the cloud, but both complemented each other. However, these algorithms did not satisfy the security and reliability of Industry 4.0 services.

To solve the abovementioned problems, the selection of the computing mode from locally or offloaded to the fog or cloud is proposed herein. The proposed algorithm minimizes the energy consumption and completion time of dependent tasks while improving the reliability of Industry 4.0 services.

## III. Preliminaries and technical background

This section introduces the three-layer system architecture and technical background of the communication mode in our system. Moreover, the computation models in the three-layer and task dependence model are presented. Table 1 presents the notations used in this paper.

**Table 1:** List of notations.

Symbol	Definition
*A*_*n*_	The computation task n attributes
b	The effective capacitance of the sensor
*C*_*n*_	The size of computation resources amount needed to perform the task n
*CT*_*n*_	The Completion time of task n
*d*_*n*_	The input data size of task n
$E_{n}^{l}$	The energy consumption for task n in the local computing
$E_{n}^{f}$	The energy consumption for task n in fog computing
$E_{n}^{c}$	The energy consumption for task n in cloud computing
$E_{n}^{exe}$	The execution energy consumption of task n
$E_{n}^{wait}$	The produced energy consumption during the waiting time for task n
$f_{n}^{l}$	The computation requirement of task n on the sensor
$f_{n}^{f}$	The computation requirement of task n on the fog
$f_{n}^{c}$	The computation requirement of task n on the cloud
*g*_*n*_	The channel gain between the sensor and the base station for the transmitting task n
*N*	The set of computing task
$P_{n}^{tra}$	The transmission power of task n
$P_{n}^{idl}$	The constant idle circuit power when the Industrial sensor is idle
Q	The optimal strategy
*r*_*n*_	The data transmission rate for task n between the sensor and the fog server through the wireless channel
$R_{n}^{fc}$	The rate of task n between the fog and the cloud in wired link
*RT*_*n*_	The ready time of task n
$T_{n}^{l}$	The total execution time for task n in the local computing
$T_{n}^{f}$	The total execution time for task n in fog computing
$T_{n}^{c}$	The total execution time for task n in cloud computing
$T_{s}^{max}$	A certain deadline constraint for service S
$T_{n}^{l}$	The total execution time for task n in the local computing
$T_{n}^{f}$	The total execution time for task n in fog computing
$T_{n}^{c}$	The total execution time for task n in cloud computing
*w*	The bandwidth of the channel between the sensor and the fog

### A. A brief system overview

A cloud system architecture is constrained because of network bandwidth limitations, the communication delay, security, and reliability. Therefore, cloud computing cannot always meet the QoS requirements of a smart factory system. Moreover, through the widespread implementation of advanced technologies, Industry 4.0 aims to improve performance, flexibility, and security in industrial automation. Thus, controller and fog-computing technologies are combined into a cloud computing system to increase the scalability and nature of the system in real time.

**Fig 2:**
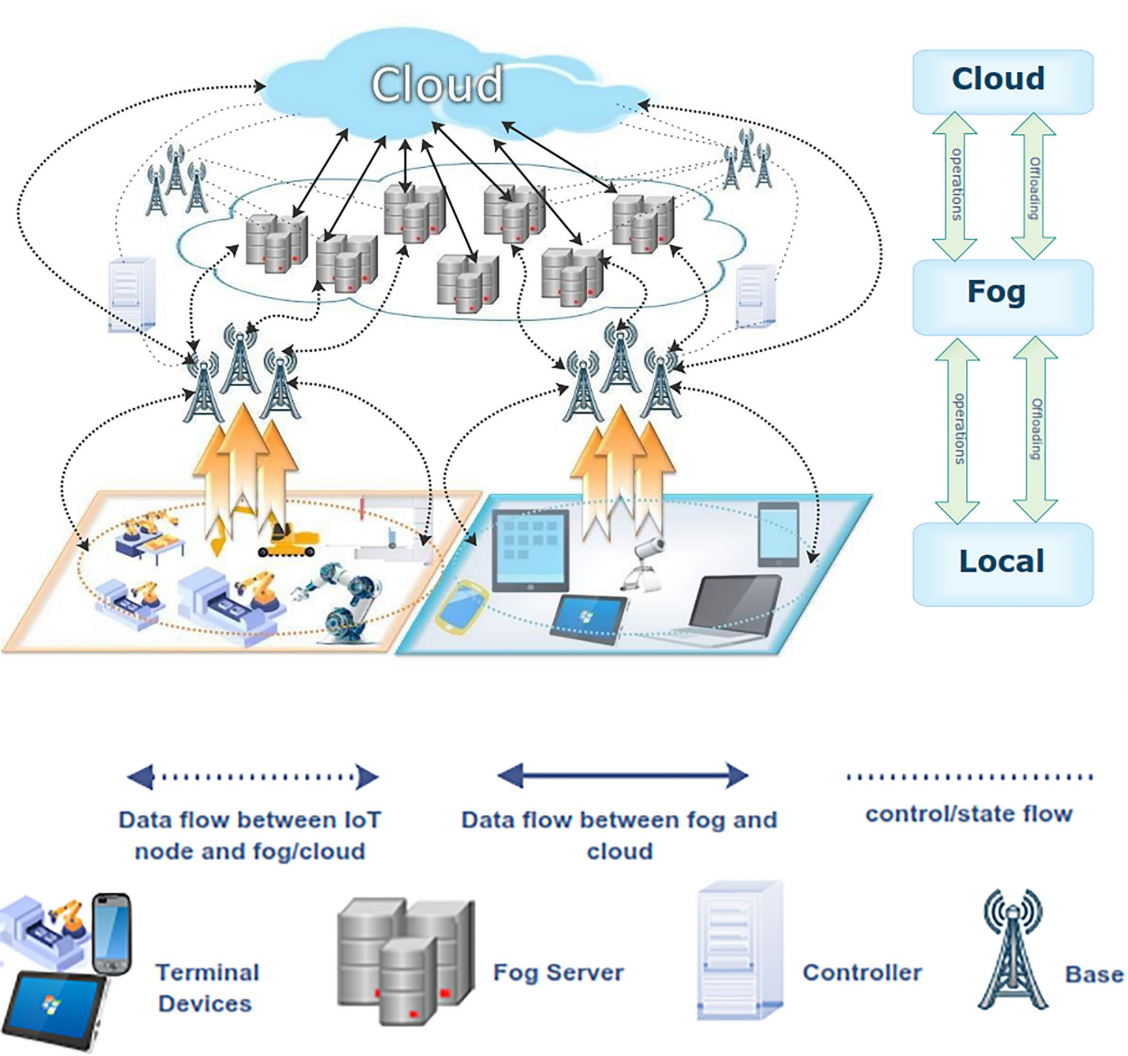
**(a)** System architecture and **(b)** legend of the system.

The system architecture can be divided into three layers: terminal devices (local computing), fog computing, and cloud computing (Fig 2). The terminal device layer is responsible for data acquisition and transmission.

This layer comprises several industrial devices, such as sensing and transmission. Each terminal device connects to the fog-computing layer responsible for processing real-time tasks on the fog servers and includes fog servers and controllers. The fog server comprises devices with limited computing capability, such as embedded servers, switches, and routers. The controller is placed to optimize the selection of the computation mode and execution sequence by managing a computation offloading process. The computation offloading is a process that collects, examines, and processes task parameters from the terminal device to fog devices or cloud servers, such as task processing and arrival rate. The controller decides whether a computing task will be executed locally or be offloaded to a fog device or cloud server. The end layer is the cloud layer responsible for handling nonreal-time and intensive-computation tasks. This layer comprises cloud storage, cloud data center, and cloud computing tool and provides remote services to the intelligent factory.

In the production line of the smart factory, *M* is a set of sensors on each terminal device, *M* = {*M*_1_, *M*_2_, *M*_3_,…,*M*_*m*_}, where *m* indicates the sensor number. *N* is the set of tasks (computation tasks) generated or requested by all the sensors on the terminal devices in the smart factory using Industry 4.0, each task *n* from sensor *m* is processed with a certain deadline constraint $T_{s}^{max}$ (in second) to satisfy QoS. The Industry 4.0 service is based on *N* computing tasks distributed among various sensors. The data dependency was considered among the computing tasks of various sensors. The set of tasks is defined as *N = {1*, *2*, *3*, *…*, *n}*. The computation task attributes are defined as *A*_*n*_ = {*d*_*n*_, *C*_*n*_, $T_{s}^{max}$}, *n*∈*N*. Where *d*_*n*_ is the input data size of a computation task *n* (in bits); *C*_*n*_ is the size of computation resources required to complete task *n* (in CPU cycles), and *C*_*n*_ depends on the computational complexity of task *n* [29]. Assuming that *A*_*n*_ was known before, the task offloading would not change through the offloading period. The main goal of this paper is to minimize the energy consumption and meet the task deadline and satisfy a well-balanced workload across all the fog and clouds in our system.

### B. Technical background

This section first introduces the communication model used in our system and then the computation models of the three layers. Finally, it presents the task dependence model.

#### 1. Communication model

The communication model adapted in our system assumes that data are sent from the sensors to the base station through a wireless link and that the base station will send the data to the fog or cloud through a wired link, such as fiber.

The processed data is returned to the base station from the fog or cloud through the same route. Thereafter, the base station returns it to the terminal device. The data transmission rate *r*_*n*,*m*_ for task *n* from sensor *m* between the terminal device and fog server through the wireless channel is calculated based on the Shannon formula [30] defined as follows:

$$r_{n,m}=w\left(1+\frac{P_{n,m}^{tra}g_{n,m}}{\sigma^{2}}\right)$$ (1)

Here, *w* is the channel bandwidth; $P_{n,m}^{tra}$is the transmission power for a task *n* from a sensor *m* (defined by the base station wireless according to the power control algorithm [31]); *g*_*n*,*m*_ is the channel gain between the sensor and base station for transmitting task *n*; and *σ*^2^ is the variance of the complex white Gaussian noise channel.

The transmission delay of task output, which is the amount of time the router takes to get a full task *n* from the sensor *m* from the input link and put the same task on the output link, is usually ignored [22,32]. Similarly, the data size after task computing is neglected because it is small, usually one-hundredth or thousandth of the task input. For example, the task output size was a few KB when the size of the task input was hundreds of KB or a few MB. Thus, only the transmission rate is considered between the sensor and fog server.

#### 2. Computation model

Suppose task *n* has an input data size *d*_*n*,*m*_ and the total number of CPU cycles *C*_*n*,*m*_ to process. Each task, as mentioned, can be processed either locally by the terminal device or offloaded to a fog device or cloud server. Thus, the computing model is discussed next.

- *Local computing*. For task computing on a local device, $f_{n,m}^{l}$ defines the computation requirements of the sensor for task *n* from sensor *m*. The local execution time and energy consumption for task *n* are defined, respectively, as follows [28]:

$$T_{n,m}^{l}=\frac{C_{n,m}}{f_{n,m}^{l}}$$ (2)

$$E_{n,m}^{l}=\gamma C_{n,m}$$ (3)

The coefficient of energy consumption per CPU cycle is defined as *γ* = *b f*^2^, where *b* is the effective capacitance of the sensor defined and is specified by the manufacture and its value ranges between 10^−11^ and 10^−27^, and *f* is the clock frequency of the chip [33].

- *Fog computing*. For task computing on a fog server, the processing of task *n* is divided into two phases. The first phase is the transmitting phase where the industrial sensor sends task data to fog through wireless transmission. The second phase is the fog-computing phase where task *n* is executed in the fog. Fog processing delay for each task is the sum of the delay due to the transmission of task data through wireless links and fog-server computing time. The total task delay and energy consumption of fog computing for task *n* are calculated as Eqs (4) and (5), respectively [28].

$$T_{n,m}^{f}=\frac{d_{n,m}}{r_{n,m}}+\frac{C_{n,m}}{f_{n,m}^{f}}$$ (4)

$$E_{n,m}^{f}=P_{n,m}^{tra}\left(\frac{d_{n,m}}{r_{n,m}}\right)+P_{n,m}^{idl}\left(\frac{C_{n,m}}{f_{n,m}^{f}}\right)$$ (5)

Where $f_{n,m}^{f}$ defines the computation requirements of the fog server for task *n* from sensor *m*, and $P_{n}^{idl}$ is the constant idle circuit power when the industrial sensor is idle.

- *Cloud computing*. If a computing task is offloaded to a cloud server, the industrial sensor first transmits its data to the base station through wireless transmission. Thereafter, the data is sent to the cloud through a wired link. Thus, the latency of the cloud processing task is equal to the sum of the delays in transmitting wireless links, transmitting wired links, and computing cloud servers. The cloud computing delay and energy consumption are determined as follows [28]:

$$T_{n,m}^{c}=\frac{d_{n,m}}{r_{n,m}}+\frac{d_{n,m}}{R_{n,m}^{fc}}+\frac{C_{n,m}}{f_{n,m}^{c}}$$ (6)

$$E_{n,m}^{c}=P_{n,m}^{tra}\left(\frac{d_{n,m}}{r_{n,m}}\right)+P_{n,m}^{idl}\left(\frac{d_{n,m}}{R_{n,m}^{fc}}+\frac{C_{n,m}}{f_{n,m}^{c}}\right)$$ (7)

Where $R_{n,m}^{fc}$ defines the data transmission rate for task *n* between the fog and cloud through the wired link, and $f_{n,m}^{c}$ is the computation requirement of the cloud for task *n* from sensor *m*.

#### 3. Task dependency model

The computation offloading strategy during task completion time is affected by data dependency among tasks. Thus, all tasks that depend on task *n* must be completed before executing task *n*.

Thereafter, the task dependency is considered in the offloading task model. The concepts of the ready and completion times of a computing task are presented below.

**Ready time** of a task *n* is described as the earliest completion time for all its dependent tasks. The task *n* ready time *RT*_*n*,*m*_ can be expressed as follows [27]:

$${RT}_{n,m}=\{{CT}_{j}^{l},{CT}_{j}^{f},{CT}_{j}^{c}\}$$ (8)

Where *pare*(*n*) is defined as a set comprising all tasks that depends on the task *n* [34].

**Completion time** of a task *n* is determined as the time taken by it to complete execution. It includes the wait and processing times of the task. The completion time *CT*_*n*,*m*_ of a task *n* can be expressed as follows [27]:

$${CT}_{n,m}={RT}_{n,m}+T_{n,m}$$ (9)

Where *T*_*n*,*m*_ defines the execution time of the task *n*. From Eqs (8) and (9), if *pare*(*n*) is empty, the task *n* is a starting node and its ready time is equal to zero. Assuming that the transmission time for the task *n* between the fog and cloud is relatively small and can be neglected, the ready time of the task *n* equals the time required to complete all its dependent tasks.

## IV. The ET-DTCO algorithm

**Fig 3:**
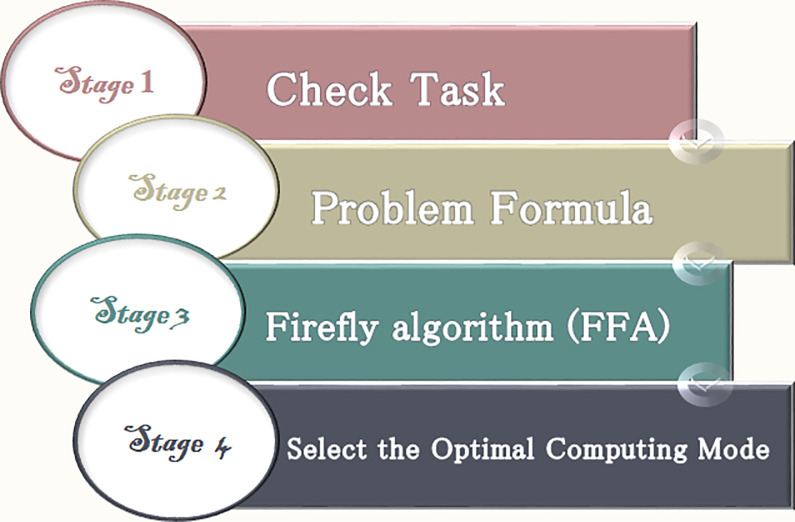
Stages of the ET-DTCO algorithm.

This section introduces the proposed ET-DTCO algorithm. The mathematical model of the optimization problem is implemented in four stages. Each stage depends on what precedes it, as shown in Fig 3.

**Fig 4:**
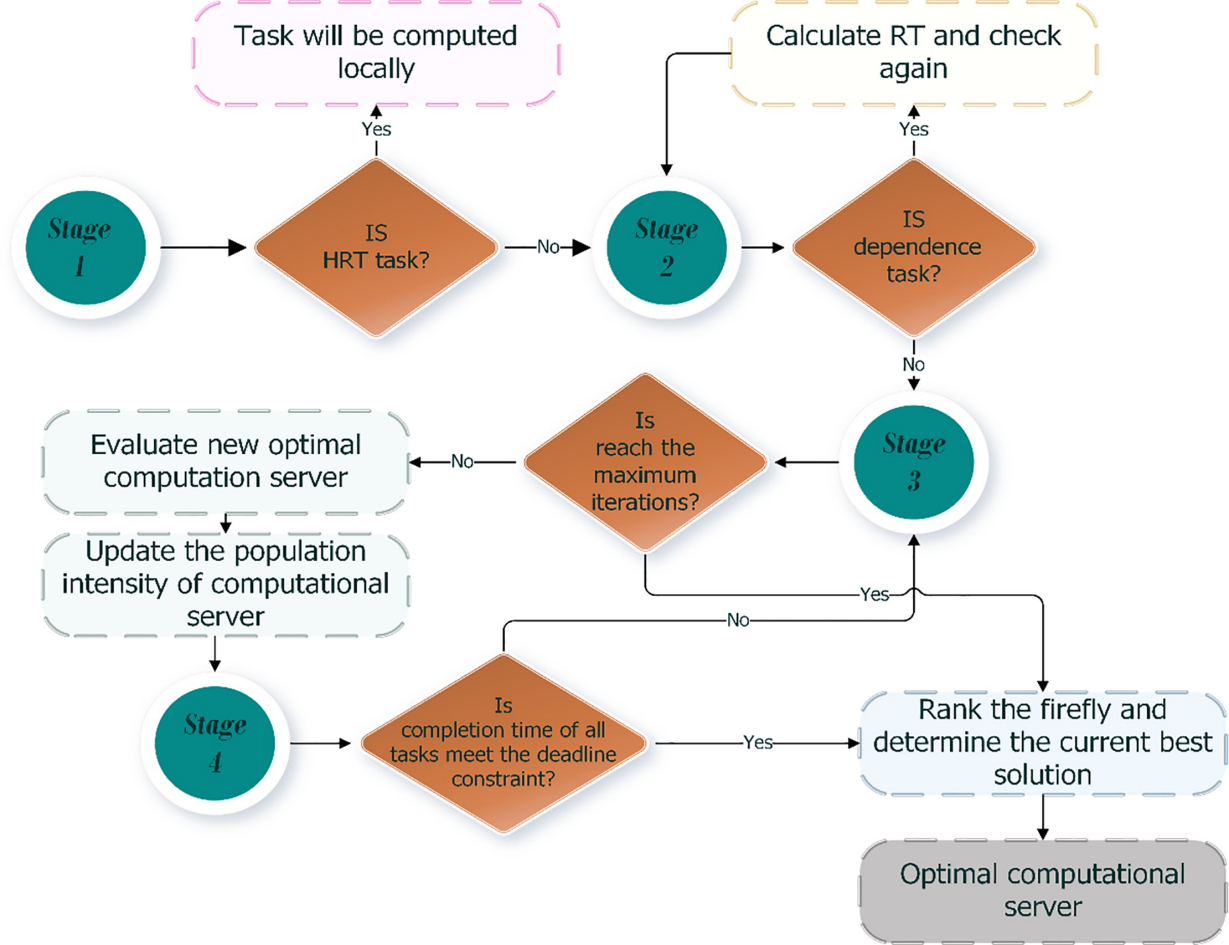
Flowchart of the ET-DTCO algorithm.

Because of avoiding the nondeterministic polynomial time NP-hard problem in our algorithm and the need to execute the computation task in a single server (fog or cloud servers) with the minimum energy and completion time, the FF algorithm [35] is used to achieve these goals and select the optimal computing server. Fig 4 shows the flowchart of the proposed ET-DTCO algorithm using FF.

Our objective offloading algorithm selects among servers similar to the FF algorithm, with updating the attractive function to calculate the attractiveness of FF (computational server) instead of distance, allowing automatically deciding the optimal offloading model using the updated objective function and brighter fireflies (optimal computational server as a type of fog or cloud servers).

### Stage 1: Check task

Factories are noisy environments, and they affect sensors and their data. Noise is anticipated; however, noise interference with the readings of sensors is unacceptable. Therefore, a task must be examined for noise before computing it. Thus, the task is compared with the factory task datasets. Thereafter, the task is ignored if found noisy; otherwise, it will be computed [36].

However, tasks are classified based on their delay tolerance. In this paper, tasks are divided into two categories; hard-real-time (HRT) and non hard-real-time (N-HRT) tasks. No delay can be tolerated by HRT tasks, and they must finish their execution within the allocated deadline. An automatic braking control system is an example of HRT tasks. A loss of life or catastrophic failure can occur if these tasks are delayed [37]. Thus, if a task is HRT and meets resource requirements, it will be locally computed; otherwise, it will be offloaded to a fog or cloud.

### Stage 2: Problem formula

Owing to the limited resource capability of terminal devices to process tasks, the terminal devices send the requests of computing tasks to fog devices (*F* = {*F*_1_, *F*_2_, *F*_3_,…,*F*_*f*_}) or cloud servers (*C* = {*C*_1_, *C*_2_, *C*_3_,…,*C*_*c*_}).

Let the computational servers be denoted by (*S* = {*S*_1_, *S*_2_, *S*_3_,…,*S*_*s*_}) where *S*∈{*F*, *C*}. According to Eqs (4) to (7), the execution energy consumption and execution time of a task *n* can be expressed as given in Eqs (10) and (11), respectively.


$$E_{n,m}^{exe}=\{E_{n,m}^{f},\;Y=1E_{n,m}^{c},\;Y=0$$ (10)

$$T_{n,m}=\{T_{n,m}^{f},\;Y=1T_{n,m}^{c},\;Y=0$$ (11)

Here, *Y* is the selection mode of the task *n* and *Y*∈{0,1}. Thus, the task *n* will be conducted either in the fog or cloud. The total energy consumption for the computation task *n* from a sensor *m* can be estimated as follows [27]:

$$E_{n,m}=E_{n,m}^{exe}+E_{n,m}^{wait}$$ (12)

Where $E_{n,m}^{wait}$ is the energy consumed during the waiting time by the task *n* due to data dependency among tasks. $E_{n,m}^{wait}$ is defined as follows [27]:

$$E_{n,m}^{wait}=P_{n,m}^{idl}\;{RT}_{n,m}$$ (13)

In this paper, we propose an offloading selection algorithm to minimize the energy consumption and task completion time of all sensors on terminal devices in an industrial IoT system by providing the optimal mode of task computation offloading. The energy consumption reduction problem can be expressed as follows (14):

$$\underset{Q}{\min}\sum_{m=1}^{M}\sum_{n=1}^{N}E_{n,m}$$ (14)

S.t:∀n∈N,∀m∈M

$$C1:E_{n,m}^{exe}\leq f_{n,m}^{exe}$$

$$C2:{CT}_{N}\leq T_{s}^{max}$$

$$C3:{RT}_{n,m}=\underset{k\in \mathrm{pare}(n-1)}{\max}{CT}_{n,m},pare(n)\neq \emptyset$$

$$C4:{RT}_{n,m}=0,pare(n)=\emptyset$$

Here, *C1* constraint indicates that the execution energy of the task must not surpass the computational offloaded mode capability. If the task is offloaded to the fog, then $f_{n,m}^{exe}=f_{n,m}^{f}$, and if the task is offloaded to the cloud, then $f_{n,m}^{exe}=f_{n,m}^{c}$.

*C*2 is the completion time constraint, indicating that the whole completion time of all the tasks must meet the deadline constraint; *C*3 and *C*4 formulate the task dependency constraints, ensuring that a task *n* is executed only if its dependent tasks are completed or if the task *n* is a starting node. According to the optimization problem in Eq (14), the FF algorithm is used to obtain an optimal strategy for this optimization problem.

### Stage 3: FF algorithm

This stage is explained in two substages; the first is the original optimization FF algorithm and the other is the proposed ET-DTCO algorithm.

#### 1. The original optimization FF algorithm

The FF algorithm is an example of swarm’s intelligence algorithms developed by XS. Yang [38]. The algorithm behavior is nature inspired by fireflies flashing behavior. Fireflies are tiny winged beetles with soft bodies having the ability to generate cold light to attract mates. Their light mechanism is similar to the capacitor mechanism wherein the charge unit limit is gradually reached and then they discharge this energy in the form of light as a signal between the sexes, mostly in flashes mating. The main purpose of the flash FF is to perform as a signal system to attract other mates. Based on FF flashing characteristics, the inspired FF algorithm has been developed to solve many complex problems in mathematics. The FF algorithm has used the following three ideal rules.

1. All fireflies are unisex; therefore, one FF uses its flashing light to attract all other mates regardless of their sex.
2. Attractiveness is proportional to the brightness of light, where a less bright FF moves to a brighter one, and inversely proportional to the distance between any two FF.
3. An FF will move randomly if it is the brightest FF, and no FF can attract it. Thus, the brightness should be determined by the objective function.

The FF algorithm is developed based on these rules to obtain the optimal solution. This algorithm comprises four important steps:

**Step 1:** In this step, the population of the FF is randomly initialized and comprises a set solution. The population is the fog devices or cloud servers in our algorithm.
**Step 2:** The distance between any two FF *i* and *j* at *x*_*i*_ and *x*_*j*_, respectively, is calculated as follows [38]:

$$v=\|x_{i}-x_{j}\|=\sqrt{\sum_{k=1}^{D}{\left(x_{i,k}-x_{j,k}\right)}^{2}}$$ (15)

Here, *D* is the optimization parameter, which is equivalent to the number of computational tasks in this study.

**Step 3:** The attractiveness of the FF, which is the objective function of our algorithm, decreases exponentially as the distance increases. The attractiveness of the FF is given by Eq (16) at a distance *v* [38].

$$\beta (v_{i,j})=\beta_{0}e^{-\gamma v_{i,j}^{2}}$$ (16)

Where *β* is the brightness of the FF at distance *v*. *β*_0_ is brightness at initial attractiveness when *v* = 0. The theoretical value of the light absorption coefficient *γ* is the variance of the attractiveness, and its value helps in determining the speed of the algorithm convergence. In most cases, the values of *γ*∈[0.01,100].

**Step 4:** In this step, the FF attraction and randomization walkthrough Levy flights. The movement of the FF is calculated based on the distance and attractiveness as follows [38]:

$$x_{i}=x_{i}+\beta_{0}e^{-\gamma v_{i,j}^{2}}(x_{j}-x_{i})+\alpha \varepsilon_{i}$$ (17)

Where *α* is the randomization variable, and *ε*_*i*_ is a random number vector derived from a Gaussian or uniform distribution. The movement of the FF is equivalent to that of the task to the fog or cloud servers in our algorithm.

#### 2. The proposed ET-DTCO fitness function

As discussed earlier, Industry 4.0 tasks may be computed locally if a task is HRT and meets resource requirements; otherwise, tasks must be offloaded to fog or cloud servers for computing and processing. To decide which computational server *S* will receive a task *n*, the minimum objective function tries satisfying two QoS parameters of the task *n*: the total energy consumption and completion time.

Each computational server (as FF) sending population intensity at location *x* and varying with distance *v* is given as follows [34]:

$${IS}_{s}(v)=IS_{0}e^{-\gamma v^{2}}$$ (18)

Here, *IS*_0_ represents the population (light) intensity of the computational server source; *S*_*s*_ is the computational server index; and *γ* is the coefficient of fixed light absorption. The light intensity for the minimization objective function is associated with the inverse of the fitness function.

Next, from Eqs (12) to (14), the task computational offloading model strategy is formulated as follows:

$$Q_{z}(n,m)=\{E_{1},E_{2},\ldots ,E_{z}\},where\;z\in \{N\}$$ (19)

This algorithm produces an objective optimization function referred to as the fitness function of the FF algorithm. The fitness function is expressed as follows:

$$fit(n,m)=Q_{z}(n)$$ (20)

The offloading objective is defined as a fitness function that determines the degree of the optimal computational server. The optimization process starts by creating a set of random solutions for finding the most suitable computational server. Producing a new solution is the sum of the current solution as expressed in Eq (17) used to generate a new solution (FF movement), where *x*_*i*_ is the new solution of the computing mode (FF), and *x*_*j*_ is the FF’s current (optimal) solution. *α*, *β*_0_, *and γ* are the control parameter values of the algorithm.

### Stage 4: Select the optimal computing mode

Each task *n* objective is standardized depending on the maximum and minimum values of the corresponding objective function when finding an optimal computational server. The standardized objective function removes the effect on multiple objectives with different amplitudes. The standardized objective is obtained as follows [38]:

$${SF}_{r}(S_{i})=\frac{f_{r}(S_{i})-f_{r}^{min}}{f_{r}^{max}-f_{r}^{min}}$$ (21)

Here, *r* represents the number of objectives, and $f_{r}^{min}\;and\;f_{r}^{max}$ represent the minimum and maximum values of the *r*^*th*^ objective, respectively. The swarm of fireflies must be ranked based on their light intensity for each generation (iteration). The FF with the highest light intensity (i.e., the solution with the minimum objective function value) is selected as a brighter one (i.e., it is a possible optimum solution), and others are revised based on Eq (17). In the last iteration, the FF with the brighter light intensity (with the minimum distance value) is selected as the brightest one (optimal solution) identifying within the swarm of fireflies.

The best-fit computational server in the system with the minimum distance value is determined as follows [38]:

$$best(S_{i})=\underset{j\neq i}{\min}\sqrt{\sum_{x=1}^{2}{\left(f_{r}(S_{i})-f_{r}(S_{j})\right)}^{2}}$$ (22)

Where the distance between *S*_*i*_ and *S*_*j*_ in two-dimensional space is defined by [38]

$$D(f_{r}(S_{i}),f_{r}(S_{j}))=\left\{ \begin{array}{l} f_{r}(S_{j})-f_{r}(S_{i})\;if\;(f_{r}(S_{j})>f_{r}(S_{i}))\\ \;0\;otherwise \end{array}\right.$$ (23)

If the solution satisfies the time constraint, the solution is returned as the optimal solution Q* = *best*(*S*_*i*_); otherwise, return to the FF algorithm and select a new solution. The proposed ET-DTCO algorithm is presented in Algorithm 1.

ALGORITHM 1: Proposed ET-DTCO algorithm

Input: task n, *w*, *σ*^2^, $T_{s}^{max}$, *C*_*n*_, *d*_*n*_, *g*_*n*_, $P_{n}^{tra}$, $P_{n}^{idl}$, $f_{n}^{l}$, $f_{n}^{f}$, $f_{n}^{c},$ *pare*(*n*), *α*, *IS*_0_, *γ*. *∀n*∈*N*.

Output: find the optimal computational

1 Compute *r*_*n*,*m*_, *CT*_*n*,*m*_, *E*_*n*,*m*_ by Eqs (1)–(13)

2 **If** *pare*(*n*) is **empty,** Then

3 *RT*_*n*_ = 0

4 **Else**

5 Calculate *RT*_*n*,*m*_, *CT*_*n*,*m*_ by Eqs (8) and (9)

6 **End if**

7 Objective function *f*(*x*) = *fit*(*n*,*m*), *x* = (*x*_1_,…,*x*_*d*_)

8 Generate initial population of fireflies *x*_*i*_(*i* = 1,2,…,*c*)

9 Light intensity *IS*_*s*_(*v*) at *x*_*i*_ is determined by Eq 18

10 Define light absorption coefficient *γ*

11 *SF*_*r*_ = InitialSolution ()

12 **While** (t < MaxGeneration) do

13 **for** *i* = 1:*s* (all s computational servers)

14     **for** *j* = 1: s (all s computational servers)

15         Calculate *SF*_*r*_(*S*_*i*_) by Eq (21)

16       **if** (*SF*_*r*_(*S*_*i*_)<*SF*_*r*_(*S*_*j*_))

17         Calculate distance between servers by Eq (23)

18             Vary attractiveness *IS*_*s*_(*v*) with distance *v* via *exp*(−*γv*)

19             Select the optimal computational server

20             Evaluate new solutions and update light intensity

21       **End if**

22     **End for j**

23    **End for i**

24   **if** (${CT}_{n,m}>T_{s}^{max}$)

25         Select another solution

26   **else**

27         Rank fireflies and find the current global best

28 **End while**

29 Post-processing the results and visualization

30 **End**

## V. Performance evaluations

For an illustration of this paper, the motivation of the proposed algorithm is based on a visit to the KAPCI coatings factory in Port Said, Egypt. This factory is one of the largest coating producers in Egypt. The smart production line in the factory needs to execute one service, such as checking if each can be appropriate and has a label, by dividing it into many sensors. Each sensor processes its task by sending several requests to the controller, which decides to execute this task locally or offloads it to the fog or cloud. In this section, simulations are performed to evaluate the proposed ET-DTCO algorithm’s performance. First, the simulation environment and used tools are presented. Thereafter, the proposed ET-DTCO algorithm’s performance is verified using the simulation results.

### A. Simulation environment

The proposed ET-DTCO algorithm’s performance was simulated and evaluated using Simu. MATLAB R2019a [39,40]. The program was implemented on an Intel Pentium i5-2450M CPU 2.50 GHz, with 8 GB RAM. The simulated industrial system was assumed to comprise 10 IoT devices, each with several IoT sensors, a total of 500 IoT sensors in the product line, 40 fogs, and 10 cloud servers. Simulation parameters are listed in Table 2. Each IoT device produces a random number of tasks per request, ranging in size from 1000 to 2000 million instructions (MI). We assume that fog and cloud nodes (computational servers) will communicate with base stations through Long Term Evolution (LTE) or a wired link. Via an LTE link, IoT devices can send task parameters (such as data size) to a controller near the base station. The controller will receive parameter and request information from the IoT device, fog, and cloud. When the controller makes its decision and chooses a computational server to process the task, the IoT device sends the task data to the base station via LTE, and the base station sends the data to the fog or cloud via wired connection. The processing results will be returned to the base station in the same way through fog and cloud. Finally, the base station sends the processing results to the appropriate IoT device. The simulation results were based on a fixed value for 100 iterations of each parameter. Moreover, the equal size tasks of various fog servers are targeting to minimize both energy and time costs.

**Table 2:** Simulation parameters.

Parameter	Value
*C*_*n*_	300 cycles/bit
*d*_*n*_	100–1000 KB uniformly
$f_{n}^{l}$	0.1–0.5 G cycles/s uniformly
$f_{n}^{f}$	2 G cycles/s
$f_{n}^{c}$	4 G cycles/s
*g*_*n*_	10^−6^
n	1:25
*N*	25
$P_{n}^{tra}$	0.1 W
$P_{n}^{idl}$	0.001–0.01 W uniformly
$R_{n}^{fc}$	5 MB/s
$T_{s}^{max}$	4 s
*w*	5 MHz
*σ*^2^	10^−9^ W
*α*	0.5:0.00001
*β*_0_	1
*γ*	0.15

**Fig 5:**
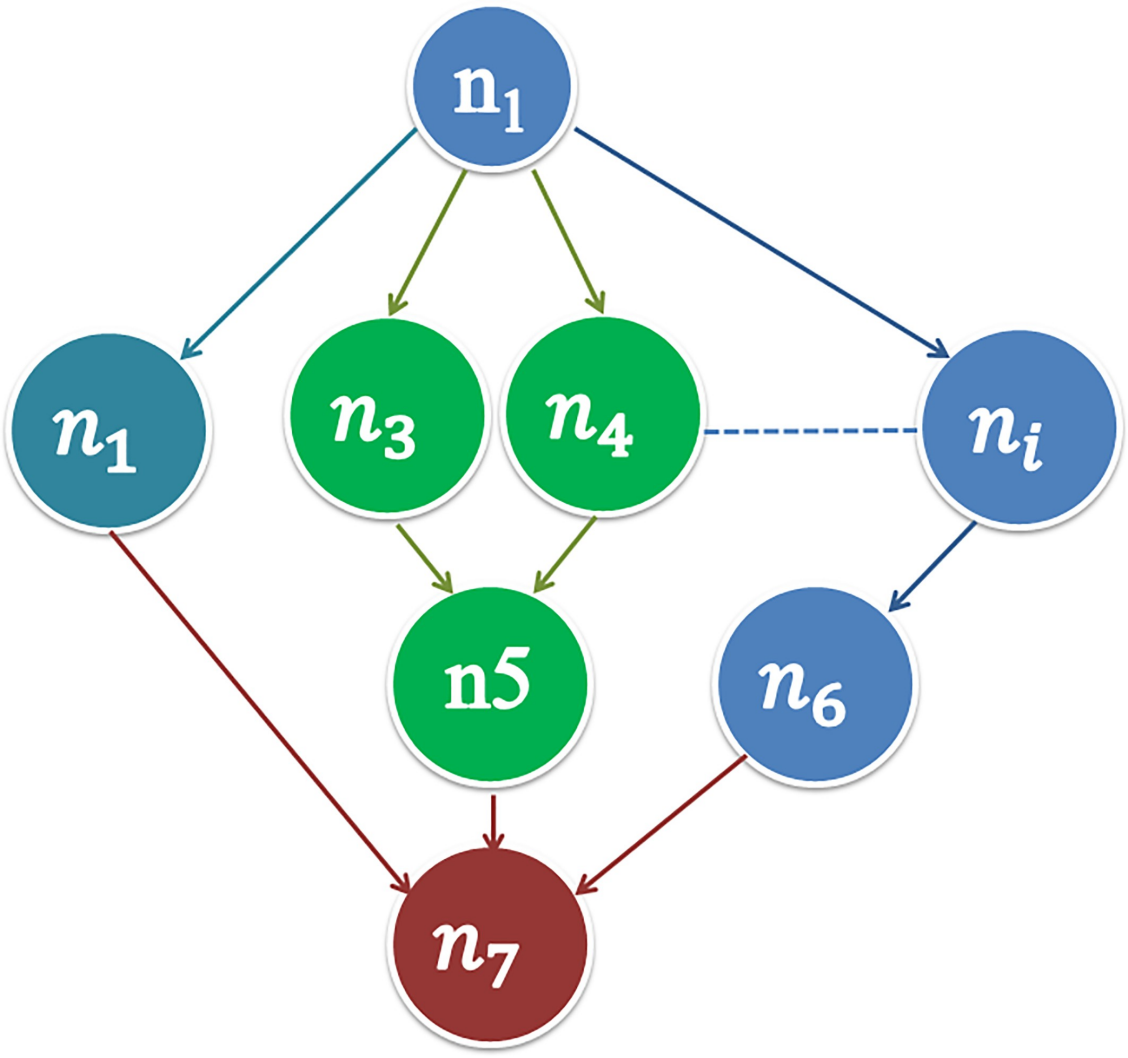
Example of the dependency relationship among tasks.

According to the task dependence model mentioned in Section III, we divided the applications into 25 tasks per sensor in the smart factory production line. Fig 5 shows an example of the relationship among the tasks. The tasks are represented as nodes from *n*_*1*_ to *n*_*i*_. The relationship among tasks is represented by the unidirectional arrow. A task cannot start execution before the previous tasks are completed. For example, the task *n*_*6*_ cannot start execution before the tasks *n*_*3*_ and *n*_*4*_ are completed, but the task *n*_*2*_ can be performed before the tasks *n*_*3*_ or *n*_*4*_ are completed.

### B. Simulation results

**Fig 6:**
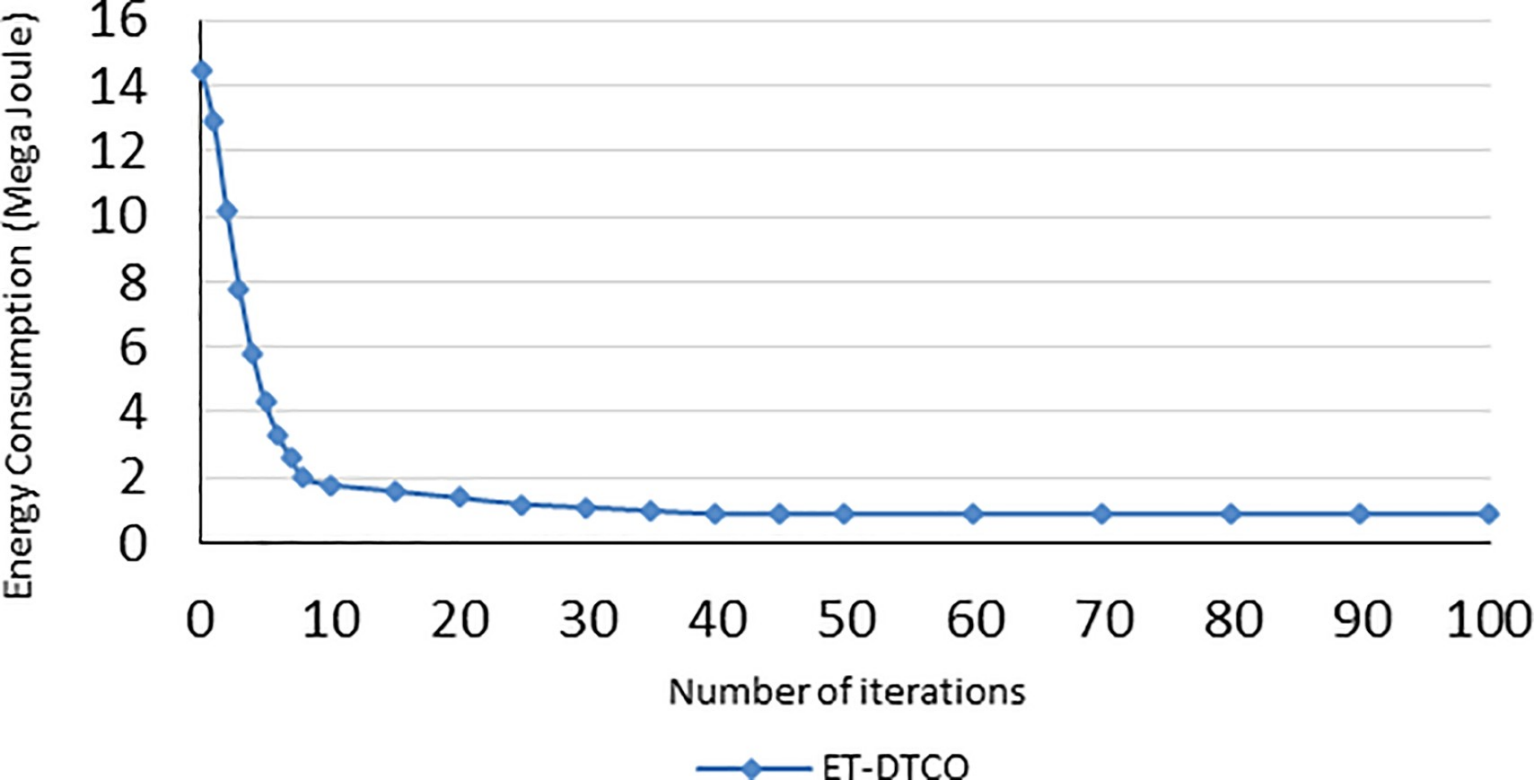
Energy consumption depends on the number of iterations.

First, we tested our system for different number of iterations *K* to determine how the number of iterations (MaxGeneration) affects the energy consumption of industrial sensors. We obtained that when the number of iterations increased, the energy consumed during the execution of the sensor tasks is decreased. The cost of the sensors dropped at the start. For example, when the number of iterations *K* was increased from 1 to 10, the sensor cost (energy consumption) decreased from 14.5 to 1.8 (MJ). Thereafter, the decrease rate slowed down with the rising number of iterations (*K* > 10) as shown in Fig 6.

**Fig 7:**
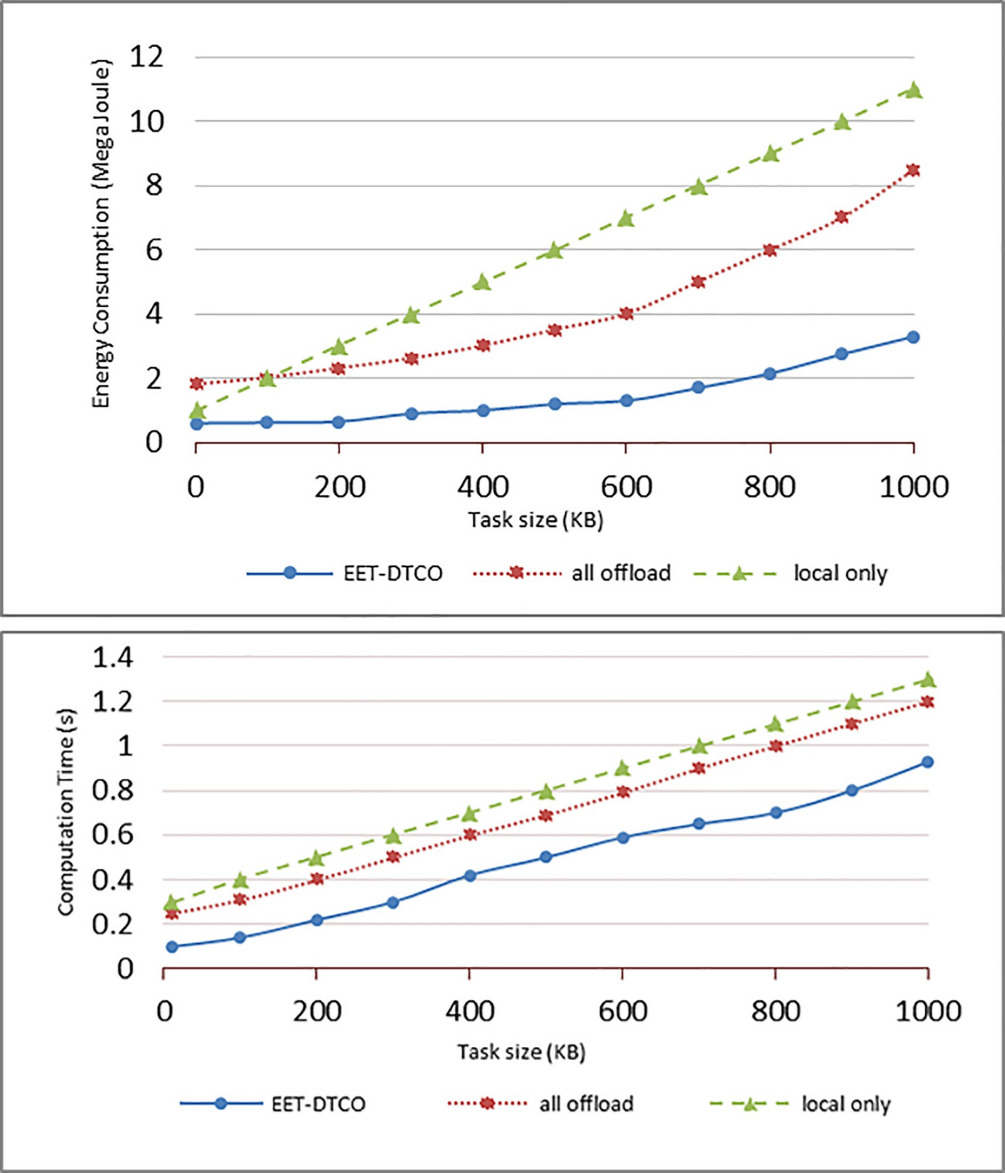
Impact of task size on **(a)** energy consumption and **(b)** computation time.

To evaluate the proposed ET-DTC algorithm’s performance, the energy consumption and computation time were calculated and compared for three scenarios. The first scenario was the case when all the tasks were offloaded to the fog or cloud. In the second case, all the tasks were executed locally. The third case was when the ET-DTCO was used. The three scenarios were tested for different task sizes ranging from 0 to 1000 KB. The total energy consumption and computation time were observed and are presented in Fig 7. Fig 7(A) and 7(B) show the impact of increasing the task size on the total energy consumed during the task execution and the total computation time for the three scenarios, respectively.

The figures show that the energy consumption and computation time increased with an increase in task size, indicating that the proposed algorithm has the lowest energy consumption and computation time. Conversely, local computing has the highest energy consumption and computation time. Although the all-task offloading algorithm consumes less energy and requires less time compared to local computing, it requires more energy and time for transmission. For example, when the task size was 500 KB, the energy consumption cost of the proposed algorithm was 1.2 MJ, and the computation time was 0.5 s. The consumed energy was lower by 65.7% and 80% compared to all-task offloading and local computing, respectively. The computation time was 7 and 8 s for all-task offload and local computing, respectively.

**Fig 8:**
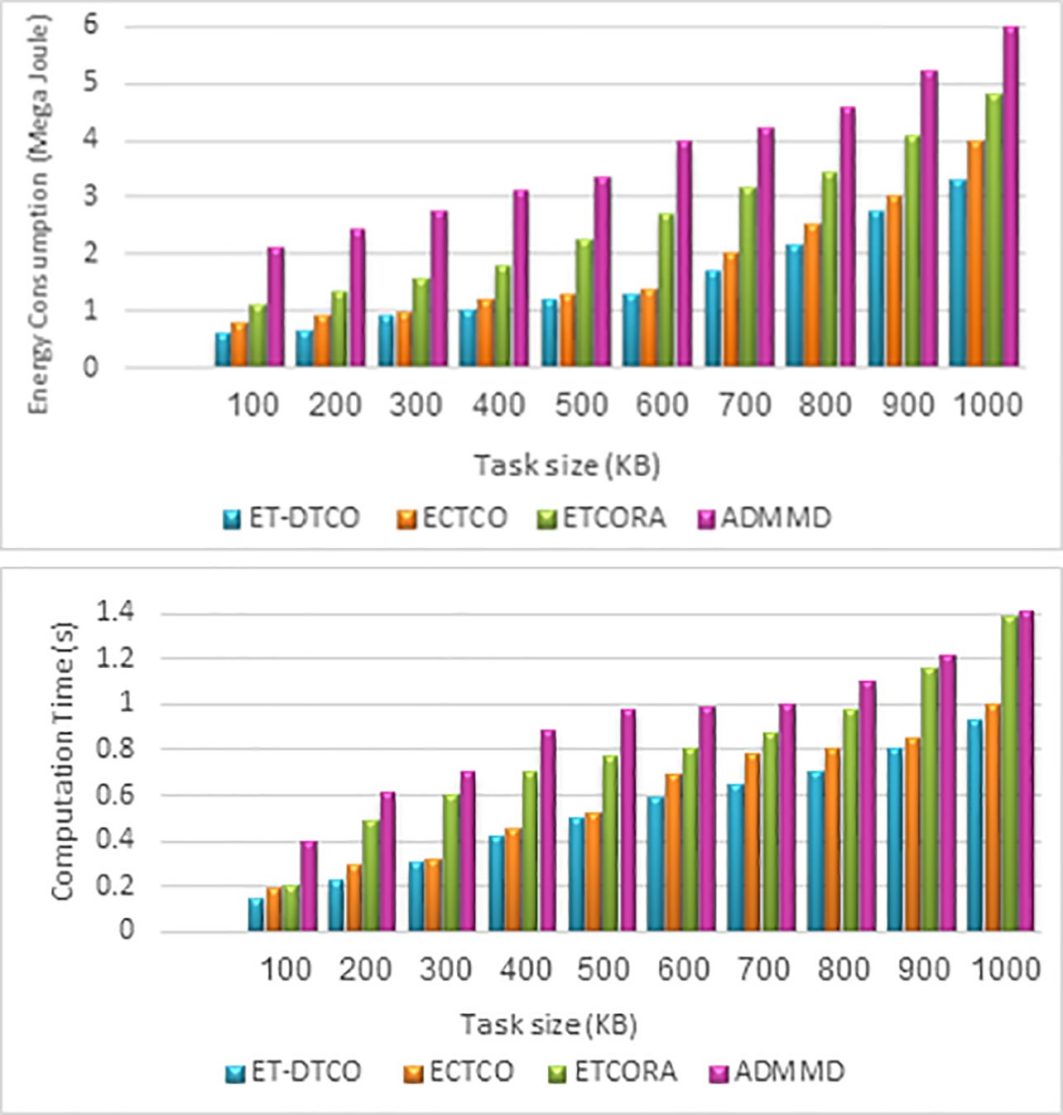
Impact of different task sizes on **(a)** energy consumption and **(b)** computation time for three algorithms.

The proposed ET-DTCO algorithm was also compared with the three existing methods, i.e., ECTCO [28], ETCORA [27], and ADMMD [26]. The energy consumption and computation time were determined for different input task sizes. Owing to the increase in the task size, the energy and time required to transmit and execute tasks increased. Consequently, the computation time and energy consumption cost of the four algorithms increased with an increase in the data size as shown in Fig 8(A) and 8(B) show the corresponding energy consumption and computation time for ET-DTCO, ECTCO, ETCORA, and ADMMD. In Fig 8(A), we can observe that ET-DTCO consumed less energy than ECTCO, ETCORA, and ADMMD for all the task sizes because they were optimized for energy consumption. However, ET-DTCO achieved the least energy consumption because it considers task dependency in Industry 4.0 applications.

During evolution, e.g., when the task size was 600 KB, the energy consumption cost of the proposed algorithm was 1.3 MJ, which was 13.2%, 53.6%, and 63.9% less than that of ECTCO, ETCORA, and ADMMD algorithms, respectively. Moreover, the computation time for the proposed ET-DTCO was less than that of the ECTCO, ETCORA, and ADMMD algorithms for all the task sizes. For example, when the task size was 500 KB, the completion time of the proposed algorithm was 0.5 s, which was 6%, 35.1%, and 45.7% less than that of the ECTCO, ETCORA, and ADMMD algorithms, respectively. The numerical results confirmed the effectiveness of the offloading strategy of the proposed ET-DTCO when compared to the existing state-of-arts- offloading algorithms in terms of reducing both the cost of energy consumption, and the computation time for dependent computational tasks.

### C. Time complexity

In this section, time complexity analysis for ECTCO, ETCORA, ADMMD and the proposed ET-DTCO algorithms are demonstrated based on number of computational servers’ population. Time complexity is the total time needed by the algorithm to run till its completion. The time complexity for ECTCO algorithm [28] was calculated by the selection of the best computational server based on *K*, a random variable of the stochastic mapping. Therefore, it is given as $O\left(K^{3.5}log\;log(\frac{1}{\epsilon })+LK\right)$, where, *ϵ* is precision parameter and *L* is the number of iteration. The time complexity of both ETCORA, and ADMMD algorithms [26,27] were calculated based on task complexity where the time complexity is increased with increasing the size of tasks. Finally, since ET-DTCO is based on FF which has time complexity that depends on the size of population (*n*) and the number of iterations (*t*) just like most other optimization algorithms. So, the overall complexity of the ET-DTCO can be expressed as *O*(*n*^2^). Based on the above complexity analysis, it is obvious that the ET-DTCO algorithm complexity is linear in terms of *t*. Therefore, the computation cost is relatively low compared to ECTCO, ETCORA, and ADMMD.

## VI. Conclusion

In this paper, a computation offloading algorithm was proposed to solve the energy consumption problem of industrial sensors in a cloud-assisted fog-computing architecture while meeting time constraints and considering the task dependency. The energy consumption problem was solved by formulating a minimization problem with deadline and task dependency constraints. The FF optimization algorithm was considered in offload decisions to select the computational server. The proposed ET-DTCO algorithm was simulated and compared with the three existing methods: ECTCO, ETCORA, and ADMMD. Moreover, the proposed algorithm was evaluated with dissimilar offloading modes. Further, it was compared with the two cases of operations: when all the tasks were offloaded to the fog or cloud and when all were executed locally. The simulation results showed that the proposed algorithm can decrease the energy consumption of industrial sensors with the constraints of the task completion deadline and dependency. The performance analysis showed that under various device parameters and dependencies, the proposed algorithm can effectively reduce the sensor cost. These simulation tests confirmed the ET-DTCO algorithm’s efficacy and adaptability.

In future works, we will apply the proposed algorithm to real-world industrial environments to conduct realistic evaluations of the proposed algorithm. Moreover, we will discuss the mobility management and issue of offloading tasks in a dynamic moving environment for sensors with inter-task dependence.

## Supporting information

S1 File Author bios. (DOCX)

S1 Dataset (XLS)
