# Supplementary material for: Efficient energy and completion time for dependent task computation offloading algorithm in industry 4.0
Source: PLoS One. 2021 Jun 8;16(6):e0252756. doi: 10.1371/journal.pone.0252756 (PMC8186806; doi:10.1371/journal.pone.0252756)
Supplement: S1 File — (DOCX) [file pone.0252756.s001.docx]

**
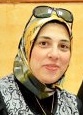
**

**Rabab Farouk Abdel-Kader** received her B.S. from the Electrical Engineering Department of Suez Canal University in 1998. She received the Ph.D. degree from the Department of Computer Science and Software Engineering at Auburn University, Auburn, AL in 2007 and the MS degree in Electrical Engineering from Tuskegee University with high honors in 2002. Since 2008 she is working as an Assistant Professor in the Electrical Engineering Department, Faculty of Engineering, Port-Said University, Egypt. Her main research interests include image processing, parallel computing, and software engineering.


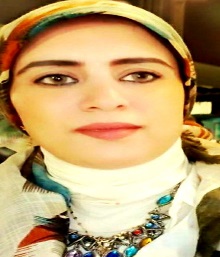


**Noha Emad El-Sayad** obtained the B.Sc. degree in computer and control engineering from Suez Canal University, in 2007. And M.Sc. degree in computer and control engineering from Port Said University, in 2014. She is currently pursuing a Ph.D. degree. She has been working as a developer in Information and Communication Technology Project (ICTP), Port Said University, Egypt, since 2012.

**
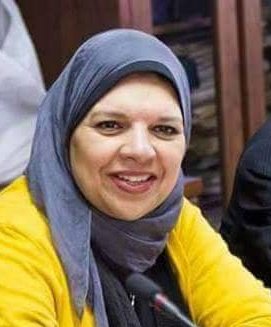
**

**Rawya Yehia Rizk** is a Professor of Computers and Control in the Electrical Engineering Department, Port Said University, Egypt. She is the Head of Electrical Engineering Department, Port Said University, 2017 till now. She is the Chief Information Officer (CIO) of Port Said University (PSU), 2014 till now. She received her BSc, MSc, and Ph.D. in Computers and Control Engineering from Suez Canal University in 1991, 1996, and 2001, respectively. Her research interest is in computer networking, including mobile networking, wireless, ATM, Sensor Networks, Ad Hoc Networks, QoS, traffic and congestion control, handoffs, and cloud computing. She was the executive director of PSU Network Infrastructure, Port Said University, 2010 till 2014. She was the manager of CISCO Academy, Faculty of Engineering, Suez Canal University, 2008 till 2010. She is the manager of CISCO Academy, Faculty of Engineering, Port Said University, 2010 till now. She is a reviewer in many of international communication and computer journals such as IEEE Access, IET communications, IET sensors, IET Networks, Journal of Supercomputing, Journal of Network and Computer Applications, Computers & Electrical Engineering, Mathematical Problems in Engineering, and IJACSA.
